# Analysis of *TGFBI* Gene Mutations in Three Chinese Families with Corneal Dystrophy

**DOI:** 10.1155/2019/6769013

**Published:** 2019-01-22

**Authors:** Feng Zhao, Yuan Liu, Tao Guan

**Affiliations:** ^1^Department of Ophthalmology, Nanjing First Hospital, Nanjing Medical University, Jiangsu, China; ^2^Department of Ophthalmology, Taizhou Municipal Hospital, Taizhou University, Taizhou, China

## Abstract

**Objective:**

To identify the types of *TGFBI* (transforming growth factor, beta-induced) gene mutations in three Chinese families with Reis–Bücklers corneal dystrophy (RBCD), lattice corneal dystrophy type I (LCDI), or Avellino corneal dystrophy (ACD) and to investigate the relationship between the phenotypes and genotypes of corneal dystrophy.

**Methods:**

Peripheral blood was collected from 24 patients and 76 phenotypically normal members in three Chinese families as well as from 100 healthy controls. Genomic DNA was extracted. All 17 exons of the *TGFBI* gene, and the exon-intron junctions were examined by polymerase chain reaction (PCR) and direct DNA sequencing to identify and analyse gene mutations. In addition, all members of the three families were subjected to detailed clinical examinations.

**Results:**

The heterozygous c.371G > T (p.R124L) mutation was detected in exon 4 of the *TGFBI* gene in nine patients from the family with RBCD. In contrast, this mutation was not found in the phenotypically normal members of the family. The heterozygous c.370C > T (p.R124C) mutation was found in exon 4 of the *TGFBI* gene in 11 patients from the family with LCDI. This mutation was not found in the phenotypically normal members of the family. The heterozygous c.371G > A (p.R124H) mutation was detected in exon 4 of the *TGFBI* gene in four patients from the family with ACD. Again, this mutation was not found in the phenotypically normal members of the family. The *TGFBI* gene mutations cosegregated with the disease phenotypes in the three families and exhibited an autosomal dominant mode of inheritance. No *TGFBI* gene mutations were detected in the 100 healthy controls.

**Conclusion:**

There is a high degree of correlation between the phenotypes and genotypes of *TGFBI*-linked corneal dystrophies. R124 represents a mutational hotspot in the *TGFBI* gene. Gene mutation analysis provides a reliable basis for the definitive diagnosis of corneal dystrophy.

## 1. Introduction

Corneal dystrophy is a general term used to describe a series of primary bilateral blinding corneal diseases that are associated with familial inheritance [1]. Currently, specific genetic backgrounds have been discovered in half of the cases of corneal dystrophy. Approximately 12 pathogenic genes have been linked to corneal dystrophy, among which the human transforming growth factor beta-induced (*TGFBI*) gene (encoding an extracellular matrix adhesion protein) is most commonly linked. It has been confirmed that the following types of corneal dystrophies are caused by *TGFBI* gene mutations: granular corneal dystrophy (GCD), Avellino corneal dystrophy (ACD), Reis–Bücklers corneal dystrophy (RBCD), and lattice corneal dystrophy (LCD). These disorders are all inherited in an autosomal dominant manner [1–5]. *TGFBI*-associated corneal dystrophies exhibit multiple clinical phenotypes and a high degree of genetic heterogeneity. Moreover, atypical variants of *TGFBI*-associated corneal dystrophy are frequently reported. Therefore, it is difficult to classify and type *TGFBI*-associated corneal dystrophies simply based on their clinical features or histological manifestations. The molecular genetic study of the pathogenic genes related to corneal dystrophies will play a positive guiding role in clinical diagnosis. In the present study, clinical characteristic analysis and molecular genetic analysis were performed on three unrelated families with different types of corneal dystrophy. The purpose of the present study was to explore the potential application of *TGFBI* gene mutation identification in the classification and diagnosis of corneal dystrophy.

## 2. Subjects and Methods

### 2.1. Subjects

**Figure 1:**
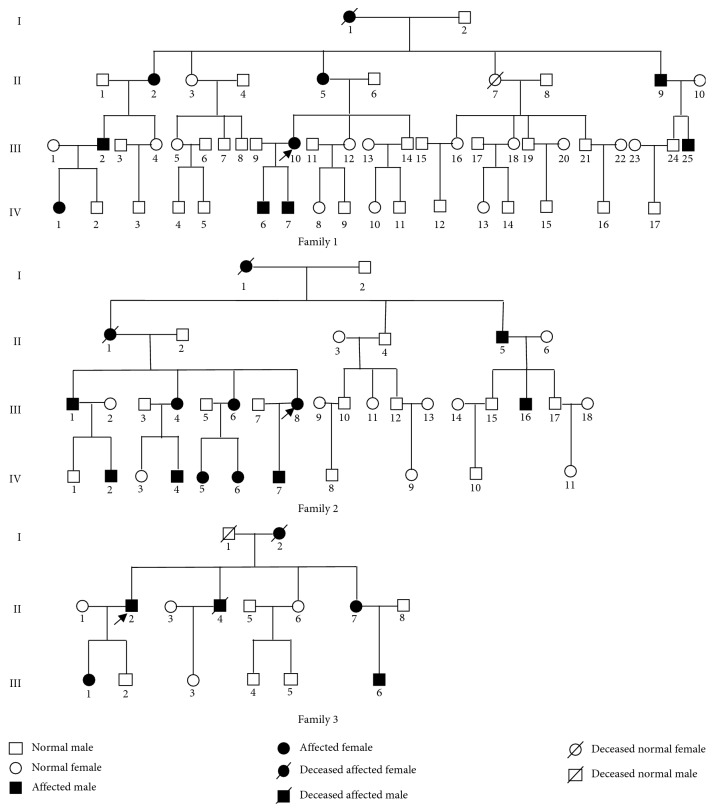
The pedigree of the three families indicates a likely autosomal dominant inheritance of Reis–Bücklers corneal dystrophy over four generations (Family 1 and Family 2) and three generations (Family 3). The genotypes and disease onset (inside parentheses) are given below the pedigree symbols. Black filled and blank symbols represent affected and unaffected status, respectively.

Three ancestrally unrelated families with corneal dystrophy that were treated in our hospital between February 2015 and October 2017 were included in the present study. In addition, 100 individuals without corneal dystrophy (confirmed by the Department of Ophthalmology) were randomly selected as controls. The study was conducted in accordance with the Declaration of Helsinki and was approved by the ethics committee of our hospital. Informed consent was obtained from all participants prior to the clinical examinations and genetic analysis. Family 1 comprised 54 members from four generations, among whom 10 members were diagnosed with corneal dystrophy (one was deceased) and 44 had a normal phenotype (one was deceased). Family 2 comprised 37 members from four generations, among whom 13 were diagnosed with corneal dystrophy (two were deceased) and 24 had a normal phenotype. Family 3 comprised 16 members from three generations, among whom six were diagnosed with corneal dystrophy (two were deceased) and 10 displayed a normal phenotype (one was deceased). The pedigrees of the three families are shown in Figure 1. The medical history of all participants as well as the patients' ages, physical signs, and symptoms were recorded. In addition, comprehensive eye examinations including refractive tests, slit-lamp microscopy, indirect ophthalmoscopy, noncontact intraocular pressure measurements, and anterior segment photography were performed to determine the phenotypes of the members of the three families.

### 2.2. Methods

#### 2.2.1. Extraction of Genomic DNA

Peripheral blood (approximately 3 mL) was collected from each of the members of the three Chinese families (including 24 patients and 76 phenotypically normal individuals) and from the 100 healthy controls. The blood samples were subjected to anticoagulant treatment with ethylenediaminetetraacetic acid (EDTA). Subsequently, genomic DNA was extracted from the blood samples using the Blood DNA Extraction Kit (Sangon Biotech Co., Ltd., Shanghai, China) according to the manufacturer's instructions. The extracted DNA was examined by 1% agarose gel electrophoresis and ethidium bromide (EB) staining. Successfully extracted genomic DNA was used as a template in subsequent assays.

#### 2.2.2. Primer Design

Primers specific for the 17 exons of the *TGFBI* gene were designed based on the available literatures [6–10] and on the DNA sequence of the *TGFBI* gene in the human genome database (http://www.ncbi.nlm.nih). The primers were synthesized by Sangon Biotech Co., Ltd. The nucleotide sequences of the primers and the polymerase chain reaction (PCR) amplification conditions are shown in Table 1.

#### 2.2.3. PCR Amplification and DNA Sequencing

An HBP220 PCR thermal cycler (Hybaid, Middlesex, UK) was used to perform PCR. Each PCR reaction mixture (50 *μ*L) contained approximately 200 ng of DNA template, 5 *μ*L of 10x buffer, 1.5 mmol/L MgCl_2_, 0.25 *μ*mol/L primers, 100 *μ*mol/L dNTPs, and 2 U of Taq polymerase (Sangon Biotech Co., Ltd.). The PCR conditions were as follows: pre-denaturation at 94°C for 5 min; 35 cycles of denaturation at 94°C for 45 s; annealing (the annealing temperatures of the various primers are shown in Table 1) for 45 s; extension at 72°C for 50 s; and a final elongation at 72°C for 10 min. The lengths of the amplified fragments are shown in Table 1. The resulting PCR products were examined by 2% agarose gel electrophoresis and EB staining. Once the PCR amplification was deemed successful, the PCR products were purified using isopropanol and were then bidirectionally sequenced using an ABI 3730 DNA analyzer (Applied Biosystems, Foster City, CA). The sequencing results were compared with the wild-type *TGFBI* gene sequence recorded in GenBank using the BLAST sequence analysis tool.

#### 2.2.4. Bioinformatic Analysis

The *TGFBI* gene mutations discovered by sequencing were assessed against the NCBI dbSNP database. In addition, SIFT (http://sift.jcvi.org/), PROVEAN (http://provean.jcvi.org/index.php), MutationTaster (http://www.mutationtaster.org/), and other online software programs were used to determine the impact of the mutations on the protein structure and function and to predict the pathogenicity of the mutations.

## 3. Results

### 3.1. Clinical Manifestations

**Figure 2:**
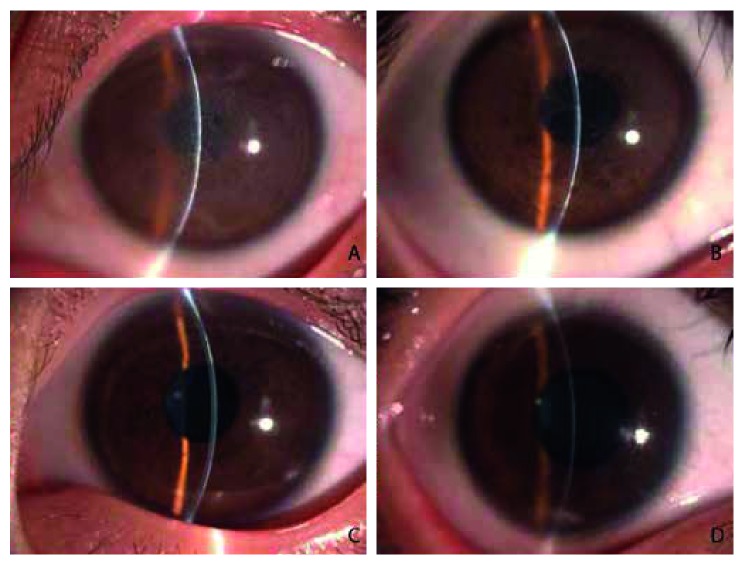
Slit-lamp biomicroscopic images of the cornea of the family with RBCD: (A) the proband (III: 10); (B) the proband's eldest son (IV: 6); (C) the proband's youngest son (IV: 7); (D) a phenotypically normal individual (IV: 8).

The proband of family 1 (III: 10) was a 36-year-old woman. She began to experience repeated irritative eye symptoms such as bilateral eye pain, red eyes, photophobia, and tearing before the age of 10. Slit-lamp microscopy revealed corneal epithelial erosion in both eyes and positive corneal fluorescein staining. In addition, the proband showed dense, map-like subepithelial opacities that involved the entire cornea (Figure 2(A)). The visual acuity of the proband deteriorated to 0.1 and could not be corrected. The eldest son of the proband (IV: 6, nine years of age) initially presented with bilateral corneal dystrophy two years before this study. The eldest son suffered noticeable corneal irritation. The subepithelial haze had spread to the periphery of the cornea (Figure 2(B)). The eldest son had a visual acuity of 0.4, which could not be corrected. The youngest son of the proband (IV: 7, six years of age) showed mild symptoms of corneal irritation. The youngest son developed irregular dot-like and sheet-like opacities in the central area of the cornea. However, the periphery of the cornea was not affected (Figure 2(C)). The visual acuity of the youngest son was 0.6, indicating that the disease was still in an early stage. Other patients in family 1 displayed clinical characteristics similar to those of the proband. Based on the clinical features, the family was preliminarily diagnosed as having RBCD.

**Figure 3:**
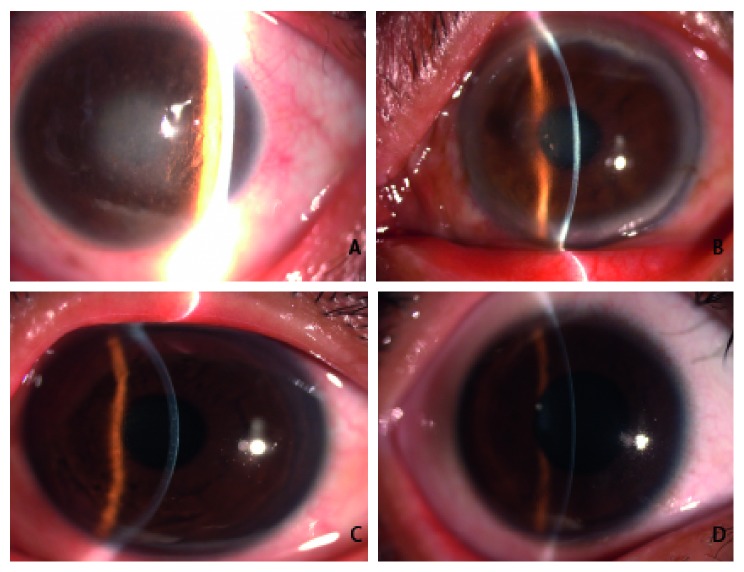
Slit-lamp biomicroscopic images of the corneas of the family with LCD: (A) the proband (III: 8); (B) the proband's son (IV: 7); (C) the youngest patient in the family (IV: 2); (D) a phenotypically normal individual (IV: 1).

The proband of family 2 (III: 8) was a 63-year-old woman. She had suffered from reduced vision for more than 30 years, and her eye condition had been worsening for three years. Her binocular visual acuity for distance was only count fingers at 30 cm (CF/30 cm), which did not improve after attempted correction. Slit-lamp microscopy revealed the presence of dense, greyish-white discoid corneal opacities in the central visual axis in both eyes. In addition, thick, lattice-shaped lesions were visible in the corneal stroma (Figure 3(A)). The proband's son (IV:7, 39 years of age) had suffered from reduced vision for more than 10 years. He had a binocular visual acuity of 0.3, which did not improve after attempted correction. The proband's son showed significantly reduced transparency in the central corneas of both eyes. Ground glass-like corneal opacities were clearly visible. In addition, semitransparent, lattice-like lesions were detected in the corneal stroma (Figure 3(B)). The youngest patient in family 2 (IV: 2) was a 16-year-old boy. The boy had a visual acuity of 0.6, which was corrected to 1.0. Nodular opacities scattered throughout the cornea were present in both eyes of the boy. In addition, thin lattice lines were found in the boy's corneal stroma (Figure 3(C)). These findings indicated that the disease was still in an early stage. Based on the clinical features, the family was preliminarily diagnosed as having LCDI.

**Figure 4:**
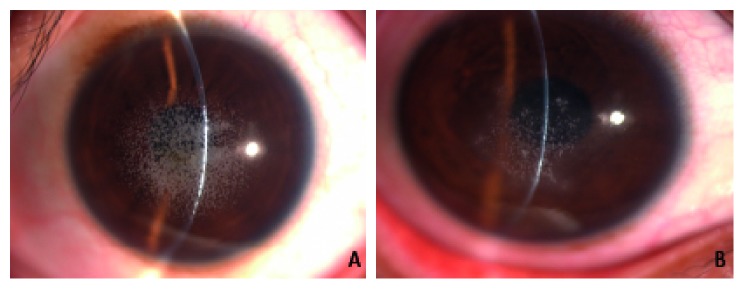
Slit-lamp biomicroscopic images of the corneas of the family with ACD: (A) the proband (II: 2); (B) the proband's daughter (III: 1).

The proband of family 3 (II: 2) was a 42-year-old male. The proband started to suffer from progressive binocular vision decline more than 10 years prior to this study without obvious cause; this decline was accompanied by a mild foreign body sensation. However, the proband did not develop apparent irritative symptoms such as eye pain, photophobia, or tearing. Slit-lamp microscopy revealed the presence of greyish-white crumb-like opacities in the superficial and middle stromal layers of the corneas in both eyes. The opacities had clear margins. The cornea between the opacities and the peripheral cornea appeared transparent (Figure 4(A)). The visual acuity of the proband was 0.2, which could not be corrected. The proband's daughter (III: 1, 19 years of age) had suffered from blurred vision for more than three years. She had a binocular visual acuity of 0.8, which did not improve after attempted correction. Greyish-white crumb-like opacities were present in the central corneas of both eyes. The opacities had clear margins. However, the density of the corneal opacities was lower in the daughter than in her father (the proband). These findings are shown in Figure 4(B). Based on the clinical features, the family was preliminarily diagnosed as having ACD.

### 3.2. DNA Sequencing

In all three families, corneal dystrophy followed an autosomal dominant mode of inheritance. Therefore, all PCR products were subjected to sequencing in both the forward and reverse directions. The results of reverse DNA sequencing were consistent with the results of forward DNA sequencing. The DNA sequencing results were compared with the *TGFBI* gene sequence in GenBank. *TGFBI* gene mutations were found in all three families, and all of the mutations occurred at R124 in exon 4 of the *TGFBI* gene (the normal codon at this position is CGC, which encodes arginine). No *TGFBI* gene mutations were detected in the 100 unrelated healthy controls.

**Figure 5:**
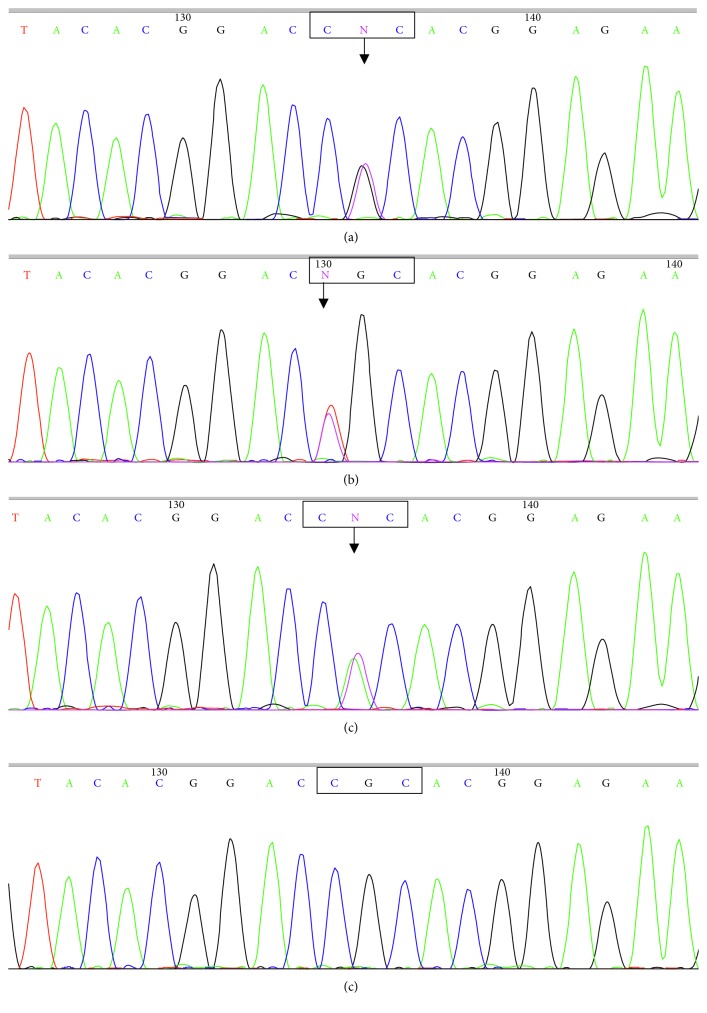
Results of the sequencing of exon 4 of the *TGFBI* gene: (a) the heterozygous G > T base substitution in family 1 resulted in a heterozygous p.R124L mutation (CGC > CTC); (b) the heterozygous C > T base substitution in family 2 resulted in a heterozygous p.R124C mutation (CGC > TGC); (c) the heterozygous G > A base substitution in family 3 resulted in a heterozygous p.R124H mutation (CGC > CAC); (d) the sequence of exon 4 in normal individuals.

The c.371G > T mutation was detected in all patients from family 1 (Figure 5(a)). This mutation was a heterozygous p.R124L mutation (CGC > CTC), in which the arginine at position 124 has been mutated to leucine. The penetrance of the p.R124L mutation was 100%. The p.R124L mutation was not detected in the phenotypically normal members of family 1. The c.370C > T mutation was detected in all patients from family 2 (Figure 5(b)). This mutation was a heterozygous p.R124C mutation (CGC > TGC), in which the arginine at position 124 has been mutated to cysteine. The penetrance of the p.R124C mutation was 100%. The p.R124C mutation was not detected in the phenotypically normal members of family 2. The c.371G > A mutation was detected in four patients from family 3 (Figure 5(c)). This mutation was a heterozygous p.R124H mutation (CGC > CAC), in which the arginine at position 124 has been mutated to histidine. The p.R124H mutation was also 100% penetrant. Again, the p.R124H mutation was not detected in the phenotypically normal members of family 3. No mutations were found in other exons of the *TGFBI* gene in the three families. Mutations at R124 of exon 4 cosegregated with disease phenotypes.

### 3.3. Results of Bioinformatic Analysis

Bioinformatic analysis showed that the p.R124L, p.R124C, and p.R124H mutations of the *TGFBI* gene had previously been reported to the NCBI dbSNP database. The effects of the above mutations on protein structure and function were assessed online using PloyPhen-2 and SIFT. The software programs predicted that all the above mutations were detrimental (Table 2). In addition, the results obtained using the MutationTaster online software indicated that the p.R124C mutation was pathogenic.

## 4. Discussion

Due to significant genetic heterogeneity, the clinical phenotypes of *TGFBI*-linked corneal dystrophy vary. The *TGFBI* gene that is located on chromosome 5q31.1 is approximately 37 kb in length and contains 17 exons. It encodes a 683 amino acid protein, namely, the corneal epithelial protein keratoepithelin (KE). KE is a secretory protein with a relative molecular mass of 68 kDa. KE contains an aminoterminal secretory signal peptide sequence, four regions of internal homology (FAS-1) consisting of 140 amino acid repeats, and a carboxyl-terminal RGD (Arg-Gly-Asp) sequence and Asp-Ile (DI) motif [6–9]. The four repeats in KE fold into a divalent tetrameric structure and act as a bridge when cells express suitable ligands. Therefore, it is believed that the TGFBI protein could mediate cell adhesion like other cell adhesion molecules. Also, TGFbeta1 has been shown to play a central role in scar formation in adult corneas. The biological effects of TGF*β* in the cornea have been shown to follow SMAD-dependent as well as SMAD-independent signaling pathways depending upon cellular responses and microenvironment. Corneal TGF*β* expression is necessary for maintaining corneal integrity and corneal wound healing [11]. It is known the molecular pathway of TGF*β* is Smad dependent. There also has evident by the fact that TGF*β* can activate several mitogen-activated protein kinases (MAPKs), including extracellular signal-regulated kinases (ERKs), c-Jun N-terminal kinases (JNKs), and p38 kinases [11, 12]. The MAPK signaling is composed of the ERK, p38, and JNK/SAPK pathways. MAPK pathways in mouse, rabbit, and human corneal epithelium mediate cell migration of corneal epithelium [13]. It has been confirmed that specific types of *TGFBI* gene mutations are highly correlated with specific clinical phenotypes of corneal dystrophy. The *TGFBI* gene mutations mainly include p.R555W (GCDI), p.R124H (ACD), p.R124L (RBCD), p.R555Q (TBCD), p.R124C (LCDI), and p.H626R (LCDI/III) [10, 14–18]. The results of the present study are consistent with these findings and confirm, once again, the strong correlation between genotype and phenotype. In addition, the results of the present study suggest that the different types of corneal dystrophy-associated *TGFBI* gene mutations exhibit no obvious regional or racial specificities. Furthermore, this study shows that the *TGFBI* gene mutations present in all patients of the three families occurred at codon R124, suggesting that this codon is a mutational hotspot in Chinese patients with *TGFBI*-associated corneal dystrophy and that mutations at R124 play important roles in the pathogenesis of this disease in Chinese patients. Future, we will further investigate the molecular mechanism involved by TGFBI mutations, like pathway.

In the present study, heterozygous mutations of the *TGFBI* gene—p.R124L, p.R124C, and p.R124H were detected in the diseased members of the three families. Bioinformatics analysis found that all these mutations were known and had detrimental effects on the structure and function of the protein encoded by the *TGFBI* gene. All patients in family 1 had the p.R124L mutation, in which hydrophilic arginine has been replaced by hydrophobic leucine. This mutation causes the deposition of corneal degeneration products and the repeated erosion of the corneal epithelium, resulting in RBCD. Clinically, visual impairment occurred early and progressed rapidly in the patients of family 1. Moreover, the patients were prone to relapse after receiving corneal transplantation. Molecular genetic analysis further demonstrated that this family had the typical map-like form of RBCD. All patients in family 2 carried the p.R124C mutation, in which the positively charged basic amino acid arginine has been replaced by the noncharged amino acid cysteine. This mutation affects the three-dimensional structure of KE, resulting in LCD. Clinically, corneal opacities and lattice-like stromal lesions were positively correlated with the age and visual impairment of the patients in family 2. Molecular genetic analysis confirmed that family 2 suffered from classic LCDI. All patients in family 3 had the p.R124H mutation, in which arginine has been replaced by histidine. Both arginine and histidine are basic amino acids; however, arginine is far more basic than histidine. The ability of arginine to form hydrogen bonds is higher than that of histidine, and arginine can effectively bind to negatively charged molecules. Replacement of arginine with histidine causes changes in the adhesion of the mutated protein to extracellular matrix proteins and in the hydrolytic activity of the mutated protein, resulting in the deposition of amyloid-like substances in the corneal stroma and ultimately causing corneal dystrophy. The discovery of the p.R124H mutation of the *TGFBI* gene in family 3 clearly demonstrated that the family suffered from ACD (i.e., GCDII).

Studies have shown that *TGFBI* gene mutations mainly affect the amino acid residue R124 and the fourth FAS-1 domain of KE. The codon R124 (which encodes arginine) is located at the amino terminus of the FAS-1 region in exon 4. Mutations at R124 may alter the polarity and hydrophilicity of the amino acid, the structure of the protein at all levels and the adhesion and hydrolysis of the protein, which induces the formation and deposition of amyloid fibrils in the corneal stroma and ultimately leads to corneal dystrophy [17, 19, 20].

In clinical practice, it is difficult to distinguish between similar phenotypes or even between subtypes of corneal dystrophies based only on the characteristics and pathological features of these corneal diseases. According to the characteristics and pathological features of corneal opacities, RBCD is classified as map-like or honeycomb-like RBCD (also known as Thiel–Behnke corneal dystrophy, TBCD) [10, 15, 17, 18]. Based on the locations of the lesions, the characteristics of the corneal opacities, and the age of onset, LCD is roughly divided into the following five types: LCDI, LCDII, LCDIII, LCDIIIA, and LCDIV. GCD has three common clinical phenotypes: GCDI, GCDII, and GCDIII. GCDII is also known as Avellino corneal dystrophy (ACD) [14–16]. At present, the classification of corneal dystrophy is rather confusing, as there are often multiple names for the same disease type. In addition, some cases of corneal dystrophy exhibit atypical clinical features. Classification and typing of corneal dystrophies based only on clinical characteristics, and histological features is difficult and is not conducive to the timely and correct diagnosis and treatment of these diseases. The examination of relevant genes will play a key guiding role in the clinical diagnosis and treatment of corneal dystrophies. Through clinical investigation and molecular genetic analysis, the present study has identified three Chinese families with corneal dystrophies caused by R124 mutations in the *TGFBI* gene and has demonstrated, once again, that R124 is a mutational hotspot in *TGFBI*-related corneal dystrophies. In addition, the present study found that the R124 mutations exhibited no regional and racial differences. However, clinical follow-up and examination of the pathogenic genes in these families will not only be conducive to prognosis prediction but will also facilitate genetic counselling and prenatal diagnoses.

There are also some limitations for this study. First, for the 24 patients, only 3 types of mutations were covered by PCR. Further, we need to use whole exon sequencing to exclude other mutations. Also, gene sequencing of all exons of the TGFBI gene should help to achieve accurate diagnosis. We only have 24 clinical patients in our study. Future, we need more samples to reveal the TGFBI gene mutation with corneal dystrophy.

## Figures and Tables

**Table 1:** Sequences of the primers specific for the 17 exons of the TGFBI gene and PCR amplification conditions.

Exon	Primer sequence (5′⟶ 3′)	Annealing temperature (°C)	Product length (bp)
1	F: CGGAGGCGCTCTCACTTCC	62	277
	R: CGAGCCCCGACTACCTGACC		

2	F: GGGAGTCATTAAAGTGGGGTGGA	64	290
	R: AGCTTGGTCTCCTGGCTGGTTAC		

3	F: CAACTTAGTGGAGAGGGGCCAGA	63	260
	R: CTCTCTCCCACCATTCCCTTCC		

4	F: GCCATCCCTCCTTCTGTCTTCTG	62	252
	R: CCTCGGGGAAGTAAGGCAGTTC		

5	F: ACTGACACCCTGTCCTTCCTCCT	67	165
	R: AGCCCACACATGGAACAGAAATG		

6	F: CTGCTCATCCTTGCTGCTTCTCT	56	248
	R: AGAGTTCCTGCTAGGCCCCTCTT		

7	F: TCTGTGGGGAGTGCCAGAGTC	55	238
	R: CAAATGAGGCAGCAGCAGGA		

8	F: TGGACCCTGACTTGACCTGAGTC	56	310
	R: AAAGGATGGCAGAAGAGATGGTG		

9	F: CCCTGGGGTGGATGAATGATAAA	62	192
	R: GCCTCCAGGGACAATCTAACAGG		

10	F: ATTGCAGGAGCACATCTCTCTGG	60	222
	R: GCTTCCCAGGAGCATGATTTAGG		

11	F: GCCCCTCGTGGAAGTATAACCAG	55	228
	R: ATCCCACTCCAGCATGACCACT		

12	F: GGGCCCTGAGGGATCACTACTTT	56	199
	R: TGACAGGTGACATTTTCTGTGTGTG		

13	F: CAGCCTTTGATTTGCAGGACACT	58	195
	R: TGACCAGGCTAATTACCATTCTTGG		

14	F: CCAACTGCCACATGAAGAAAAGG	60	280
	R: TGCTCTACCTTTCAACCACTACTCTG		

15	F: CCTCTATGGCCCAAACAGAGGAC	57	231
	R: TACCTCTGGTCAAACCTGCCTTT		

16	F: ATACAGCAGATGGCAGGCTTGG	55	246
	R: GCCATTGTCATAAGCAGTTGCAG		

17	F: ATTGAGGTGTGGAGGAGCATGAC	62	214
	R: TGGGGAGATCTGCACCTATTTGA		

**Table 2:** Analysis of the effects of TGFBI gene mutations on protein function using different software programs.

Type of mutation	PolyPhen-2 software		SIFT software		MutationTaster software
	Score assessment		Score assessment		Assessment
p.R124L	0.965	Detrimental	0.01	Detrimental	Pathogenic
p.R124C	1	Detrimental	0.01	Detrimental	Pathogenic
p.R124H	0.999	Detrimental	0.03	Detrimental	Pathogenic

All the software programs including PolyPhen-2, SIFT, and MutationTaster predicted that the three mutations (p.R124L, p.R124C, and p.R124H) of the *TGFBI* were detrimental and pathogenic.

## Data Availability

The data used to support the findings of this study are available from the corresponding author upon request.
